# ALS Genetics, Mechanisms, and Therapeutics: Where Are We Now?

**DOI:** 10.3389/fnins.2019.01310

**Published:** 2019-12-06

**Authors:** Rita Mejzini, Loren L. Flynn, Ianthe L. Pitout, Sue Fletcher, Steve D. Wilton, P. Anthony Akkari

**Affiliations:** ^1^Centre for Molecular Medicine and Innovative Therapeutics, Murdoch University, Perth, WA, Australia; ^2^The Perron Institute for Neurological and Translational Science, Perth, WA, Australia; ^3^Centre for Neuromuscular and Neurological Disorders, The University of Western Australia, Perth, WA, Australia

**Keywords:** amyotrophic lateral sclerosis, TDP-43, FUS, missing heritability, disease mechanisms, cell models, therapeutics

## Abstract

The scientific landscape surrounding amyotrophic lateral sclerosis (ALS) continues to shift as the number of genes associated with the disease risk and pathogenesis, and the cellular processes involved, continues to grow. Despite decades of intense research and over 50 potentially causative or disease-modifying genes identified, etiology remains unexplained and treatment options remain limited for the majority of ALS patients. Various factors have contributed to the slow progress in understanding and developing therapeutics for this disease. Here, we review the genetic basis of ALS, highlighting factors that have contributed to the elusiveness of genetic heritability. The most commonly mutated ALS-linked genes are reviewed with an emphasis on disease-causing mechanisms. The cellular processes involved in ALS pathogenesis are discussed, with evidence implicating their involvement in ALS summarized. Past and present therapeutic strategies and the benefits and limitations of the model systems available to ALS researchers are discussed with future directions for research that may lead to effective treatment strategies outlined.

## Introduction

Amyotrophic lateral sclerosis (ALS) is a fatal motor neuron disease characterized by degenerative changes in both upper and lower motor neurons (Rowland and Shneider, 2001). Onset typically occurs in late middle life and presents as a relentlessly progressive muscle atrophy and weakness, with the effects on respiratory muscles limiting survival to 2–4 years after disease onset in most cases (Chio et al., 2009). ALS is the most common adult motor neuron disease with an incidence of 2 per 100,000 and prevalence of 5.4 per 100,000 individuals (Chiò et al., 2013). Current treatment options are based on symptom management and respiratory support with the only approved medications in widespread use, Riluzole and Edaravone, providing only modest benefits and only in some patients (Petrov et al., 2017; Sawada, 2017). Many factors have contributed to the slow progress in developing effective treatments for this devastating disease. Although ALS is believed to have a large genetic component with high heritability, many of the gene variants that cause or predispose an individual to develop ALS remain unknown. Furthermore, with so many cellular processes implicated in ALS disease progression, determining which are causative remains challenging. The complex nature of the disease and large genetic and phenotypic heterogeneity between patients also complicates matters, making it difficult for studies in genetically similar animal models to translate to success in human clinical trials.

This review discusses the genetic landscape of ALS in the context of ALS research and reviews disease mechanisms and cellular pathways implicated in ALS disease progression. Past and current therapeutic strategies and model systems available to ALS researchers are also explored and the factors contributing to the slow progress in therapeutic development discussed.

## Genetics of Als

Evidence from clinical and basic research suggests multiple causes of ALS, with important but varied genetic components. Up to 10% of ALS affected individuals have at least one other affected family member and are defined as having familial ALS (fALS); almost all of these cases have been found to be inherited in an autosomal dominant manner (Kirby et al., 2016). The remaining 90–95% of ALS cases occur in people with no prior family history; these individuals are said to have sporadic ALS (sALS) (Chen et al., 2013).

**FIGURE 1:**
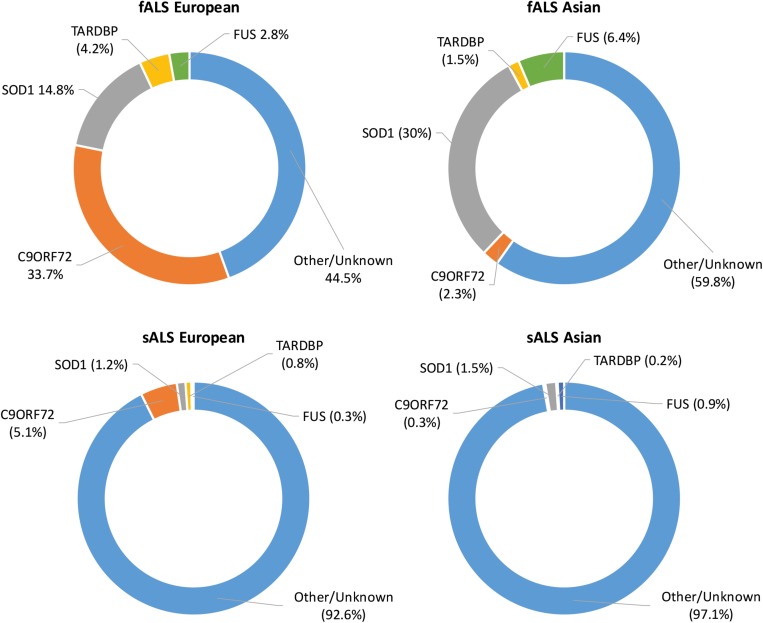
Proportion of ALS explained by the four most commonly mutated genes in Asian and European populations. Data adapted from Zou et al. (2017).

As technology has advanced, molecular genetic techniques have been increasingly applied to ALS research. Genome-wide association studies and “next-generation” sequencing techniques have supplemented the “first generation” methods, such as genetic linkage analysis, and have allowed the search for ALS-linked genes to be conducted in large sample sets (Boylan, 2015). Such advances have contributed to our understanding of the genetic causes of fALS with approximately 40–55% of cases accounted for by variants in known ALS-linked genes (Zou et al., 2017). Although more than 50 potentially causative or disease-modifying genes have been identified, pathogenic variants in *SOD1*, *C9ORF72*, *FUS*, and *TARDBP* occur most frequently with disease causing variants in other genes being relatively uncommon (Boylan, 2015). The proportion of ALS cases attributed to variants in the most common ALS-linked genes in European and Asian populations can be seen in Figure 1. In sALS cases however, diagnostic advancements have only helped in explaining a fraction of cases, with the etiology remaining unexplained in over 90% of patients (Renton et al., 2014). Genetic risk factors are widely considered to contribute to sALS with estimates from twin studies putting heritability at around 60% (Al-Chalabi et al., 2010). Despite many genetic association studies being carried out, the identification of heritable genetic risk factors in sALS remains elusive.

### Missing Heritability in ALS

There are several factors that may contribute to the missing heritability in ALS, including limitations due to technical issues as well as the inherently complex nature of the disease(s). One potentially large contributing factor to the missing heritability in ALS has been the limitations of the technologies used in large association studies. ALS association studies have relied extensively upon short read, high throughput sequencing technologies (Fogh et al., 2014; Wouter van et al., 2016). Although useful for detecting single-nucleotide polymorphisms (SNPs), the majority of structural variations (SVs) occurring in the human genome are not well characterized by single short-read platforms. Technical issues involving amplification of repetitive DNA regions, short-read mapping and SV detection algorithms have made SVs difficult to analyze, especially those consisting of long repeats or which occur in repetitive DNA regions (Chaisson et al., 2014; Cameron et al., 2019). Genome-wide association studies now commonly make use of genotyping arrays that cover a million SNPs across the genome and include thousands of clinically diagnosed patients. Although this method has successfully identified several novel and rare ALS-linked genes, a vast gap still exists in identifying heritable genetic traits that contribute to ALS risk. The missing heritability suggests that SNPs are unlikely to account for a substantial proportion of the genetic contribution to ALS and highlights the urgent need for alternative approaches to aid in understanding ALS etiology, including the search for genetic modifiers and risk factors.

Structural variations remain underexplored potential genetic modifiers of ALS and other diseases. Structural variations include all genomic deletions, insertions, inversions and microsatellites, with over 7 million classified in the human genome. Many of these are associated with a simple sequence repeat or tandem repeat in which a short base-pair motif is repeated several times in tandem. Simple sequence repeats have long been known to have a propensity for slippage mutations in which the number of repeats is increased or decreased during replication (Sutherland and Richards, 1994; Kovtun and McMurray, 2008). Simple sequence repeats occur frequently throughout eukaryotic genomes with the polymorphisms they produce being exploited in various applications, including lineage analysis and DNA fingerprinting. Many SVs, including short sequence repeats, are located within introns of genes leading early researchers to believe they were genetic “junk” with no effect on phenotype. The subsequent discovery of at least 20 neurological disorders caused by expanded short sequence repeats (including the *C9ORF72* expansion seen in ALS) has illuminated a pathological extreme that points to a wider influence of short sequence repeats on normal brain function (Fondon et al., 2008).

A recent study has highlighted the extent and cryptic nature of SVs when using high throughput sequencing; by utilizing a suite of long-read and short-read sequencing technologies and several SV calling algorithms, a three to sevenfold increase in the detection of SVs was reported, compared to standard high throughput sequencing, with an average of over 800,000 SVs uncovered per genome (Chaisson et al., 2018). Furthermore, on a per variant basis SVs are 50 times more likely to affect the expression of a gene compared with a SNP (Chiang et al., 2017). Polymorphic structural variants may account for differences between individuals at risk for a specific phenotype, including the risk of disease, disease course, mechanism of pathogenesis and response to treatment, particularly in complex genetic diseases such as ALS (Roses et al., 2016). The characterization of SVs that occur within and around regulatory regions of ALS associated genes may reveal a source of novel sALS heritability or shed light on risk factors or pathogenic mechanisms.

There are several other factors that may contribute to the missing heritability in ALS, including the potential oligogenic nature of the disease. The difficulty in uncovering the genetic determinants of ALS suggests the disease has a complex genetic architecture that may consist of combinations of gene variants differing in frequency and noxiousness. Interest in the potential oligogenic nature of ALS has recently increased. The number of ALS patients reported to have more than one ALS risk variant varies depending on the study, but has been reported to be between 1 and 4% (Kenna et al., 2013; Cady et al., 2015; Morgan et al., 2017). Some studies have reported that the presence of a second risk variant further increased the risk of ALS and disease progression (Pang et al., 2017). Others have suggested that those with a known highly penetrant risk allele are more likely to be defined as being oligogenic than controls for a given panel of variations, introducing an often uncontrolled bias to these studies. A recent study on oligogenic variation in neurodegenerative disease found that after controlling for the major known variant there was no association between the second likely benign oligogenic variation and neurodegenerative disease (Keogh et al., 2018). Complicating matters further, what counts as a secondary variant is debatable, as many variants are of uncertain significance. Low frequency variants could account for a substantial part of the missing heritability in ALS. Variants of low minor allele frequency may not be captured by current genotyping arrays and effect sizes may not be large enough to be detected by linkage analysis in families (Manolio et al., 2009). Additionally, the small number of patients harboring some potentially deleterious variants can make determining pathogenicity difficult. Substantially larger datasets than those studied to date will be required to resolve this issue.

The way that genetic diagnoses are made may also be contributing to the apparent missing heritability in ALS. Genetic diagnosis is often done by whole exome sequencing, resulting in potentially important intronic and intergenic variants being missed. An increase in rare variants, many of unknown significance, has been found in the untranslated regions of known disease-causing genes including *SOD1, TARDBP, FUS, VCP, OPTN* and *UBQLN2*, highlighting the potential importance of regulatory gene regions when determining disease pathogenesis and making genetic diagnoses (Morgan et al., 2017). Heritability can also be hard to determine in some cases due to the incomplete penetrance of variants in many ALS-associated genes. *C9ORF72* and *ATXN2* variants for example show incomplete penetrance, with symptoms not always manifesting in mutation carriers (Elden et al., 2010; Murphy et al., 2017). This may lead to inherited cases that appear to be sporadic. Monogenic high penetrance variants may therefore account for a large proportion of those with no apparent family history (Al-Chalabi and Lewis, 2011). Several social and clinical factors come into play when determining whether disease is familial or sporadic, with the distinction looking increasingly artificial.

A large proportion of the genetic risk for sALS remains elusive; this has meant much research to date has focused on understanding how variations and differences in expression of known ALS-linked genes lead to disease. *SOD1*, *TARDBP*, *FUS*, and *C9ORF72* have been most extensively characterized.

## Als Associated Genes

### SOD1

The *SOD1* gene (encoding superoxide dismutase 1 [Cu/Zn]) was the first to be associated with ALS, in 1993 (Rosen et al., 1993). *SOD1* encodes a 153 amino acid metalloenzyme, one of three superoxide dismutase enzymes found in humans. The protein binds copper and zinc and forms an extremely stable homodimer. SOD1 dimers reside in the cytosol and the intermembrane space of mitochondria, providing an important antioxidant defense mechanism by catalyzing the production of oxygen and hydrogen peroxide from the superoxide species produced during cellular respiration (McCord and Fridovich, 1969). A recent meta-analysis found that pathogenic variants in *SOD1* account for approximately 15–30% of fALS and fewer than 2% of sALS cases (Zou et al., 2017).

Over 185 disease-associated variations in *SOD1* have now been identified and are distributed throughout the gene (Yamashita and Ando, 2015). The majority are missense mutations, with the D90A variant the most common worldwide. Phenotype, disease duration and severity can differ significantly depending on the variants involved. Rapid disease progression and shorter survival times are seen in patients with the A4V, H43R, L84V, G85R N86S, and G93A variants, whilst patients with the G93C, D90A, or H46R variants generally have longer life expectancies (Yamashita and Ando, 2015). Genotype-phenotype correlations in SOD1-ALS are apparent with distinct clinical features manifesting in patients harboring particular variants. The A4V variant, for example, is associated with a limb-onset, aggressive ALS form (Juneja et al., 1997). Patients homozygous for the D90A variant generally display a slowly progressive paresis that starts in the legs and gradually spreads upward, as well as some atypical features such as bladder disturbance (Andersen et al., 1996). In contrast, heterozygous D90A variation is associated with several ALS forms including bulbar, upper limb onset and lower limb onset with a faster progression (Hong-Fu and Zhi-Ying, 2016).

#### SOD1 Disease Mechanisms

Variations in *SOD1* have been associated with a decrease in enzyme activity of 50–80% (Deng et al., 1993; Rosen et al., 1993), leading to early propositions that disease was conferred through a loss of dismutase activity. However, a later study showed that dismutase activity did not correlate with disease severity, indicating that a toxic gain of function mechanism might be at play (Cleveland et al., 1995). Support for a toxic gain of function mechanism was soon supported by a *Sod1-*knockout mouse model that did not display an ALS phenotype (Siwek et al., 1996). Mutation-induced conformational and functional changes of SOD1 have been proposed to confer toxicity via interactions with many proteins and through several mechanisms. These include excitotoxicity, oxidative stress via upregulation of reactive oxygen species, endoplasmic reticulum stress, mitochondrial dysfunction, and prion-like propagation as reviewed by Hayashi et al. (2016). As in other neurodegenerative diseases involving protein aggregation, debate surrounds whether the soluble or aggregated forms of the protein are responsible for exerting toxicity. Additionally, non-native formations of wild-type SOD1 have been detected in small granular SOD1-immunoreactive inclusions in the motor neurons of sALS patients without pathogenic *SOD1* variants (Forsberg et al., 2010) and in patients carrying the *C9ORF72* repeat expansion and pathogenic variants in other ALS-associated genes (Forsberg et al., 2019). This suggests that misfolding of wild-type SOD1 may be deleterious or be part of a common downstream event in ALS progression.

### TDP-43

Histological examinations of spinal cord samples had revealed that neuronal cytoplasmic ubiquitinated inclusions were present in the majority of ALS patients (Leigh et al., 1991). In 2006, a shift in the understanding of ALS pathogenesis occurred with the discovery that the main component of the ubiquitinated protein aggregates found in sALS patients was TAR DNA-binding protein 43 (TDP-43) (Arai et al., 2006; Neumann et al., 2006). Further histological studies have since confirmed that TDP-43 is present in the cytoplasmic aggregates of the majority of ALS patients including sporadic cases without pathogenic variants in the *TARDBP* gene, and in those with *C9ORF72* hexanucleotide repeat expansions (Giordana et al., 2010; Schipper et al., 2016; Takeuchi et al., 2016). The aggregation of TDP-43 in ubiquitin-positive cytoplasmic neuronal inclusions in the brain and spinal cord is now considered a pathological hallmark of ALS.

**FIGURE 2:**
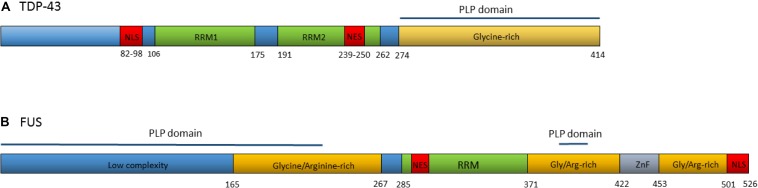
**(A)** Domain structure of TDP-43. Numbers refer to amino acid positions. NLS is nuclear localization signal, NES is nuclear export signal, RRM1 and RRM2 are RNA recognition motifs. Prion-like protein (PLP) domain spans from amino acids 274–414. ALS associated mutations are clustered in the glycine-rich region. **(B)** Domain structure of the FUS protein. Numbers refer to amino acid positions. NLS is nuclear localization signal, NES is nuclear export signal, RRM is the RNA recognition motif, ZnF is the Zinc-Finger domain. Prion-like protein domains span from amino acids 1–239 and 391–407. A cluster or ALS associated mutations occur within the nuclear localization signal with others spread throughout the gene.

TDP-43 is a DNA/RNA binding protein composed of 414 amino acids, encoded by the *TARDBP* gene. Although usually concentrated in the nucleus, TDP-43 contains both a nuclear localization signal and a nuclear export signal (Figure 2A) and shuttles back and forth between the nucleus and cytoplasm (Ayala et al., 2008). TDP-43 functions as a regulator of gene expression and is involved in several RNA processing steps with roles in pre-mRNA splicing, regulation of mRNA stability, mRNA transport, translation, and the regulation of non-coding RNAs (Buratti and Baralle, 2010; Tollervey et al., 2011; Ratti and Buratti, 2016).

In 2008, dominant mutations in the *TARDBP* gene were identified as a primary cause of ALS, providing evidence that aberrant TDP-43 could be causative of neurodegeneration (Gitcho et al., 2008; Kabashi et al., 2008; Sreedharan et al., 2008; Van Deerlin et al., 2008; Yokoseki et al., 2008). To date, at least 48 variants in *TARDBP* have been associated with ALS (Lattante et al., 2013). The majority of these are missense mutations located in the glycine-rich region at the carboxy-terminal of the transcript. The carboxy-terminal region interacts with other heterogeneous ribonucleoproteins and is involved in pre-mRNA splicing regulation (Buratti et al., 2005; Neumann et al., 2006).

#### TDP-43 Disease Mechanisms

The cytoplasmic accumulation of TDP-43 is concomitant with a loss of nuclear TDP-43; this has led to proposed mechanisms of disease involving a loss of normal TDP-43 function in the nucleus, a toxic gain of function, or both. Various animal models have been generated to test the loss of function hypotheses. Homozygous TDP-43 null mice are not viable, demonstrating that TDP-43 is vital in embryonic development (Kraemer et al., 2010; Sephton et al., 2010; Wu et al., 2010). An inducible knockout in adult mice also proves lethal (Chiang et al., 2010). Mice heterozygous for *TARDBP* deletion displayed motor deficits but no degeneration of motor neurons and no reduction in TDP-43 protein levels (Kraemer et al., 2010).

Much of the evidence for the gain of function hypothesis comes from overexpression models. TDP-43 overexpression rodent models have consistently found that overexpression of both wild-type and mutant TDP-43 can cause a neurodegenerative phenotype (Ash et al., 2010; Kabashi et al., 2010; Liachko et al., 2010; Stallings et al., 2010; Wils et al., 2010; Xu et al., 2011). Overexpression of normal human TDP-43 in mouse models can cause fragmentation of the protein, resulting in the production of the signature 35 and 25 kDa fragments seen in human ALS cases (Wils et al., 2010).

**FIGURE 3:**
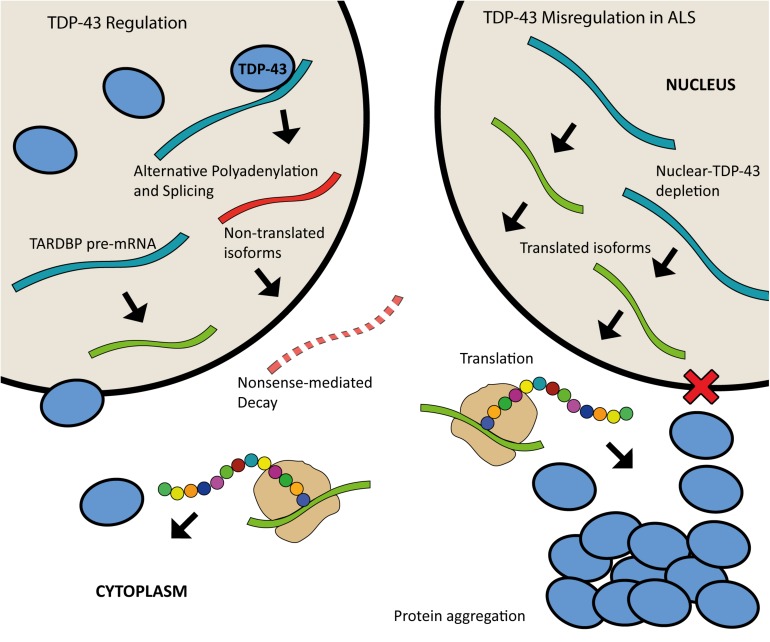
Schematic of TDP-43 autoregulation and misregulation in ALS. When in nuclear excess, TDP-43 binds to a region in the 3′UTR of the TARDBP pre-mRNA leading to alternative polyadenylation and splicing; this produces mature mRNA transcripts that are subject to nonsense mediated decay. Nuclear depletion of TDP-43 as seen in ALS (right) leads to continuous upregulation of TDP-43 synthesis as more of the translated isoforms are produced.

Both the loss and overexpression of TDP-43 are causative of disease, highlighting the importance of tightly controlled regulation of this protein. There is increasing evidence that ALS may be caused by aberrant TDP-43 regulation. Expression of TDP-43 is autoregulated through a feedback mechanism by which the protein binds to a region within the 3′UTR of its own pre-mRNA when in nuclear excess, triggering the use of alternative polyadenylation signals and splicing events that result in mRNA transcripts that are degraded rather than translated (Avendaño-Vázquez et al., 2012; D’Alton et al., 2015; Koyama et al., 2016). The depletion of TDP-43 from the nucleus is thought to result in the continuous upregulation of TDP-43 synthesis (Figure 3) (Koyama et al., 2016). TDP-43 homeostasis is critical for normal cellular function. Excess TDP-43 in the cytoplasm may result in the formation of inclusion bodies leading to cellular dysfunction whilst nuclear depletion may induce widespread dysregulation of mRNA metabolism, with TDP-43 knockdown shown to lead to the differential splicing or expression of hundreds of targets (Highley et al., 2014; Colombrita et al., 2015; Klim et al., 2019).

In addition to the abnormal distribution and aggregation of TDP-43 in ALS, several post-translational modifications are associated with pathologic TDP-43, including ubiquitination, proteolytic cleavage, and phosphorylation. There is also evidence of less well characterized post-translational modifications of TDP-43 that include acetylation, sumoylation, disulfide bridge formation and others, as reviewed by Buratti (2018). The temporal sequence of these post-translational modifications and the role each plays in disease onset remains unclear. There is also accumulating evidence that ALS may share similarities to prion-like disorders in which the accumulation of misfolded proteins is self-propagating. Disease propagation can be seen clinically in ALS as a focal onset of disease that spreads to neighboring sites (Ravits et al., 2007; Brettschneider et al., 2013). Such a mechanism may be operating in ALS aided by the presence of TDP-43’s prion-like protein domain (Nonaka et al., 2013; Mompeán et al., 2016).

### FUS

In 2009, pathogenic variants in the gene encoding another RNA-binding protein, fused in sarcoma (FUS), were identified in a subset of ALS patients (Kwiatkowski et al., 2009; Vance et al., 2009). *FUS* variants are associated with early onset and juvenile ALS (Zou et al., 2013; Hübers et al., 2015a, b; Gromicho et al., 2017). FUS-ALS is characterized by pathological FUS aggregation, generally reported to occur only in patients with pathogenic variants in the *FUS* gene. TDP-43 aggregation is not commonly seen in FUS-ALS patients suggesting that the FUS disease pathway is independent of TDP-43 (Vance et al., 2009).

*FUS* encodes a ubiquitously expressed 526 amino acid protein belonging to the FET family of RNA binding proteins. FUS is predominantly localized to the nucleus under normal physiological conditions but crosses over to the cytoplasm, functioning in nucleocytoplasmic transport (Zinszner et al., 1997). FUS shares many physiological roles with TDP-43; playing a role in several aspects of gene expression including transcription, pre-mRNA splicing, RNA transport and translation regulation (Ratti and Buratti, 2016). Although they share many similarities, TDP-43 and FUS regulate different RNA targets and show different sequence binding specificity (Lagier-Tourenne et al., 2012; Colombrita et al., 2015). Additionally, FUS is involved in DNA repair mechanisms including both homologous recombination during DNA double-strand break repair and in non-homologous end joining (Baechtold et al., 1999; Mastrocola et al., 2013; Wang W.-Y. et al., 2013). FUS also plays a role in the formation of paraspeckles providing cellular defense against various types of stress (Hennig et al., 2015).

Over 50 autosomal dominant *FUS* variants have now been identified in ALS patients. The majority are missense mutations, although in rare cases insertions, deletions, splicing, and nonsense mutations have been reported (Lattante et al., 2013). Many of the pathogenic variants are clustered within the nuclear localization signal and lead to the redistribution of FUS to the cytoplasm (Niu et al., 2012; Vance et al., 2013). Others occur in the glycine and arginine-rich regions, the prion-like domain and the 3′UTR (Figure 2B) (Shang and Huang, 2016). Variants within some regions appear to increase the propensity of the protein to form solid aggregates, pointing to various pathomechanisms operating in FUS related ALS (Nomura et al., 2014).

#### FUS Disease Mechanisms

As with TDP-43, debate continues as to the extent to which a loss of function or a gain of function mechanism causes disease in FUS-ALS. FUS loss of function theories suppose that the pathologic cytoplasmic redistribution of FUS renders it incapable of carrying out its functions in the nucleus. A homozygous *Fus* knockout mouse model was developed in 2015, and while the mice survived into adulthood, they did not show ALS-like phenotypes, suggesting that loss of FUS is not sufficient to cause ALS (Kino et al., 2015). This result was followed by development of further models in which FUS was conditionally removed from the motor neurons of mice, with no significant loss of motor neurons or denervation (Sharma et al., 2016). However, the findings were contradictory when Drosophila *Fus* knockdown models were used, whereby neuronal degeneration and locomotive defects followed the knockdown of the *FUS* ortholog *Cabeza* (Sasayama et al., 2012).

There is strong evidence for gain of function mechanisms operating in FUS-ALS. A transgenic mouse model overexpressing wild-type human FUS reportedly developed an aggressive phenotype of motor neurodegeneration and evidence of cytoplasmic FUS accumulation (Mitchell et al., 2013). There is debate as to whether toxicity is primarily mediated by the FUS aggregates directly or via an increase in soluble FUS in the cytoplasm after its redistribution. There is some evidence that toxicity may be caused by FUS aggregates directly. Cytoplasmic FUS accumulation and a severe motor phenotype were observed in transgenic mouse models that express aggregate-prone FUS variants lacking the ability to recognize and bind RNA (Shelkovnikova et al., 2013; Robinson et al., 2015). Additionally, as seen with TDP-43, there are indications of a propagating mechanism of disease in FUS-ALS, possibly mediated by its prion-like protein domain (Lee and Kim, 2015; Feuillette et al., 2017). There is also evidence that soluble cytoplasmic FUS may be toxic. Several rodent models in which neurodegeneration was observed have displayed increased cytoplasmic FUS, without the formation of aggregates (Huang et al., 2011; Scekic-Zahirovic et al., 2016; Sharma et al., 2016). Cytoplasmic FUS distribution also alters stress granule dynamics (Vance et al., 2013; Lenzi et al., 2015). Some have proposed that FUS aggregation may be a compensatory mechanism protecting cells from potentially toxic increases in soluble cytoplasmic FUS. Rather than purely pathologic, the propensity of FUS to aggregate is important in normal cellular functions (Hennig et al., 2015). Impaired cellular function can also be the direct result of pathogenic *FUS* variants that have been reported to cause splicing defects, DNA damage and to compromise FUS autoregulation (Wang W.-Y. et al., 2013; Zhou Y. et al., 2013; Qiu et al., 2014; Rulten et al., 2014).

### C9ORF72

In 2011, the most common inherited cause of ALS in European populations, a hexanucleotide repeat expansion (GGGGCC) in the non-coding region of the *C9ORF72* gene, was discovered (DeJesus-Hernandez et al., 2011; Renton et al., 2011). Typically, 5–10 copies of this hexanucleotide repeat expansion are present in the gene, but ALS patients with the expansion may have hundreds to thousands of repeats. This hexanucleotide repeat expansion occurs in approximately 34% of fALS and 5% of sALS cases in European populations, but is less frequent in Asian populations (Zou et al., 2017). The function of the *C9ORF72* product is poorly understood, but recent reports have pointed toward functions in regulating endosomal trafficking and autophagy (Farg et al., 2014; Nassif et al., 2017). Several *C9ORF72* deficient mouse models have shown immune dysregulation pointing to a possible function of C9ORF72 in the immune system (Atanasio et al., 2016; Burberry et al., 2016).

#### C9ORF72 Disease Mechanisms

The *C9ORF72* mRNA and protein levels were reportedly decreased in ALS patients with repeat expansions, leading to the hypotheses that a loss of the protein may be implicated in disease (Waite et al., 2014; Xiao et al., 2015). A *C9ORF72* conditional knockout mouse model was generated to test this hypothesis but the animals were not found to show evidence of motor neuron degeneration or motor deficits, indicating that a loss of C9ORF72 function alone was insufficient to cause motor neuron disease (Koppers et al., 2015). Furthermore, the targeted knockdown of *C9ORF72* RNA is well tolerated in mice and in ALS patient induced pluripotent stem cell (iPSC) derived neurons (Donnelly et al., 2013; Lagier-Tourenne et al., 2013). It is therefore supposed that loss of *C9ORF72* function alone is not a major cause of C9 related frontotemporal dementia or ALS.

There is evidence for several toxic gain of function mechanisms underpinning C9ORF72-ALS. The G rich nature of the repeats makes the transcript susceptible to the formation of highly stable G-quadruplex secondary structures that trigger abnormal interactions with a range of proteins (Fratta et al., 2012). Although numerous pathways may be misregulated in C9ORF72-ALS, RNA misprocessing has consistently appeared as a key pathway affected. Firstly, processing of the expanded transcript itself is altered. The C9ORF72 transcript contains two alternatively used first exons (1a and 1b) with the repeat expansion residing in the intron between them. The various isoforms produced from the transcript contain one or both of the exons with the presence of the repeat expansion favoring transcription from exon 1a. This leads to an increase in the proportion of transcripts produced that contain the repeat expansion (Sareen et al., 2013). Other misregulated RNA processing events affecting the repeat containing transcripts have also been described including abortive transcription, decreased splicing of the repeat containing intron and nuclear aggregation (Barker et al., 2017). Additionally, a portion of the repeat-containing transcripts are subject to repeat-associated non-ATG (RAN) translation, resulting in the production of abnormal dipeptide repeat proteins that also form neuronal inclusions in the CNS and may contribute to disease pathogenesis via various mechanisms (Ash et al., 2013; Gendron et al., 2013). Furthermore, the expanded repeat causes several downstream effects on RNA processing. RNA transcripts containing the repeat expansion form discrete nuclear structures referred to as RNA foci (DeJesus-Hernandez et al., 2011) that are found in the majority of C9ORF72-ALS patients (Schipper et al., 2016). Toxicity may arise as these RNA foci are able to aberrantly interact with and sequester various RNA-binding proteins, impairing their function and leading to more general effects on RNA expression and splicing (Todd and Petrucelli, 2016). Consensus has not yet been achieved on the extent to which each of these mechanisms contributes to disease progression.

### Genes With Minor Involvement in ALS

The increasing use of next-generation sequencing of family pedigrees as well as large cohorts of ALS patients has led to the discovery of rare genetic variants associated with ALS in many other genes, as shown in Table 1. These variants have been found primarily in genes that influence RNA processing, protein homeostasis and cytoskeletal dynamics. Although rare, they provide hints as to some of the pathogenic mechanisms underpinning ALS. The link between a particular gene variant and disease is not always clear due to the small number of patients harboring some of the variants, as well as study size limitations leading to inconclusive results. For example, several studies have reported that DNA variants in the paraoxonase locus are associated with sALS (Saeed et al., 2006; Landers et al., 2008; Valdmanis et al., 2008). However, these findings were not replicated when a larger genome wide meta-analysis was undertaken (Wills et al., 2009).

**TABLE 1:** Genes thought to be causative of ALS.

**Involved in**	**Gene**	**Protein**	**Functions include**	**References**
RNA processing	TARDBP	TAR DNA-Binding Protein, 43-Kd	Splicing regulation, RNA transport, miRNA biogenesis	Rutherford et al., 2008; Sreedharan et al., 2008; Neumann, 2009
	FUS	Fused in sarcoma	Splicing regulation, RNA transport, maintenance of genomic integrity, miRNA processing	Kwiatkowski et al., 2009; Vance et al., 2009
	ANG	Angiogenin	RNA processing, neurite outgrowth, vascularisation, stress granule formation	Greenway et al., 2006; Wu et al., 2007
	SETX	Senataxin	DNA/RNA metabolism and helicase activity	Chen et al., 2004; Hirano et al., 2011
	MATR3	Matrin 3	RNA processing, chromatin organization	Johnson et al., 2014b
	ATXN2	Ataxin 2	RNA processing (interacts with TDP-43), endocytosis, modulates mTOR signaling	Elden et al., 2010; Sproviero et al., 2016
	TAF15	TATA-binding protein-associated factor 2N	transcription initiation; RNA polymerase II	Couthouis et al., 2011; Ticozzi et al., 2011
	EWSR1	EWS RNA Binding Protein 1	RNA splicing, transcriptional repressor,	Couthouis et al., 2012
	hnRNPa1	Heterogeneous nuclear ribonucleoprotein A1	mRNA processing, splicing, and transport	Kim et al., 2013; Naruse et al., 2018
	hnRNPA2B1	Heterogeneous nuclear ribonucleoproteins A2/B1	mRNA processing, splicing, and transport	Kim et al., 2013; Fifita et al., 2017
	ELP3	Elongator complex protein 3	Protein synthesis, maturation of projection neurons	Simpson et al., 2009
Protein Trafficking and degradation	C9ORF72	Guanine nucleotide exchange C9orf72	Transcription, splicing regulation, endosomal trafficking, autophagy	DeJesus-Hernandez et al., 2011; Renton et al., 2011
	ALS2	Alsin	Endosomal dynamics and trafficking, neurite outgrowth	Hadano et al., 2001; Yang et al., 2001
	VAPB	Vesicle-associated membrane protein-associated protein B/C	Vesicle trafficking	Nishimura et al., 2004; Sanhueza et al., 2013
	CHMP2B	Charged multivesicular body protein 2b	Multivesicular body formation, protein trafficking to lysosomes	Parkinson et al., 2006; Cox et al., 2010
	FIG4	Polyphosphoinositide phosphatase	Endosomal trafficking to Golgi network, autophagy regulation	Chow et al., 2009; Osmanovic et al., 2017; Bertolin et al., 2018
	UBQLN2	Ubiquilin-2	Protein degradation via UPS	Deng et al., 2011; Gellera et al., 2013
	SQSTM1	Sequestosome-1 (p62)	Protein degradation via UPS and autophagy	Fecto et al., 2011; Rubino et al., 2012; Hirano et al., 2013
	SIGMAR1	Sigma non-opioid intracellular receptor 1	Lipid transport from ER, mitochondrial axonal transport, BDNF and EGF signaling	Luty et al., 2010; Al-Saif et al., 2011; Ullah et al., 2015
	OPTN	Optineurin	Golgi maintenance, membrane trafficking, exocytosis, autophagy	Maruyama et al., 2010; Tümer et al., 2012; Pottier et al., 2018
	VCP	Valosin Containing Protein	Protein degradation via UPS, autophagy, membrane fusion	Forman et al., 2006; Johnson et al., 2010
	TBK1	Tank Binding Kinase 1	Autophagy, innate immunity signaling	Borghero et al., 2015; Cirulli et al., 2015; Oakes et al., 2017
Cytoskeletal and axonal dynamics	DCTN1	Dynactin subunit 1	Axonogenesis, microtubule anchoring, ER to Golgi transport, spindle formation, vesicle transport, cilia formation	Münch et al., 2004, 2005; Takahashi et al., 2008; Liu et al., 2014
	PFN1	Profilin 1	Cytoskeletal signaling, regulates actin polymerization	Wu et al., 2012; Smith et al., 2015
	SPG11	Spatacsin	Cytoskeletal stability, regulating synaptic vesicle transport	Orlacchio et al., 2010; Daoud et al., 2012
	TUBA4A	Tubulin α-4A chain	Component of microtubules	Smith et al., 2014; Pensato et al., 2015
	NEFH	Neurofilament heavy polypeptide	Maintenance of neuronal caliber, intracellular transport	Figlewicz et al., 1994; Al-Chalabi et al., 1999
	PRPH	Peripherin	Cytoskeletal protein, neurite elongation, axonal regeneration	Gros-Louis et al., 2004; Leung et al., 2004; Corrado et al., 2011
	NEK1	NIMA (Never In Mitosis Gene A)-Related Kinase 1	Cilia formation, DNA-damage response, microtubule stability, neuronal morphology, axonal polarity	Brenner et al., 2016; Kenna et al., 2016; Nguyen et al., 2018; Shu et al., 2018
Mitochondria	CHCHD10	Coiled-Coil-Helix-Coiled-Coil-Helix Domain Containing 10	Mitochondrial protein, cristae morphology, oxidative phosphorylation	Bannwarth et al., 2014; Chaussenot et al., 2014; Johnson et al., 2014a
	C19ORF12	Protein C19orf12	Mitochondrial protein	Deschauer et al., 2012
Other	SOD1	Superoxide dismutase [Cu-Zn]	Cytosolic Antioxidant	Rosen et al., 1993
	ERBB4	Receptor tyrosine-protein kinase erbB-4	Neuronal cell mitogenesis and differentiation	Takahashi et al., 2013
	SS18L1	Calcium-responsive transactivator	Neuron specific chromatin remodeling	Teyssou et al., 2014
	PNPLA6	Neuropathy target esterase	Regulation of neuronal membrane composition	Rainier et al., 2008
	PON1-3	Paraoxonase 1-3	Enzymatic breakdown of nerve toxins	Saeed et al., 2006; Wills et al., 2009; Ticozzi et al., 2010
	DAO	D-amino-acid oxidase	Regulates D-serine levels, N-methyl-D-aspartate receptor regulation	Mitchell et al., 2010; Morgan et al., 2017
	CHRNA3,4,B4	Neuronal acetylcholine receptor subunit α-3, α-4, β-4	Cholinergic Neurotransmission	Sabatelli et al., 2009, 2012
	ALS3	ALS3	Disulfide redox protein	Hand et al., 2002
Unknown	ALS7	Unknown	Unknown	Sapp et al., 2003
	ALS6-21	Unknown	Unknown	Butterfield et al., 2009
	ALS-FTD	Unknown	Unknown	Dobson-Stone et al., 2013

In addition to the large number of gene variants thought to be primary causes of ALS, a number of variants appear to influence ALS phenotype or susceptibility. Common variants of the *UNC13A* gene, for example, have been associated with susceptibility to ALS and shorter survival time of patients (Diekstra et al., 2012). Intermediate length trinucleotide repeat expansions of the both the *ATXN1* and *ATXN2* genes also increase the risk of disease, particularly so for *C9ORF72* expansion carriers in the case of *ATXN1* (Sproviero et al., 2016; Lattante et al., 2018). A copy number variation of the *EPHA3* gene, in contrast, has been flagged as a potential protective factor for ALS (Uyan et al., 2013). Many other potential genetic modifiers of ALS risk have been identified, including variations in genes implicated in detoxification pathways, highlighting the potential interplay of environmental and genetic factors (Dardiotis et al., 2018). As well as gene variants, variations in gene expression can also alter ALS disease susceptibility or phenotype. Although no association was found between any of the 654 SNPs occurring in the *EPHA4* gene and ALS susceptibility, disease onset and survival inversely correlated with EPHA4 expression (Van Hoecke et al., 2012).

## Proposed Mechanisms of Disease

**FIGURE 4:**
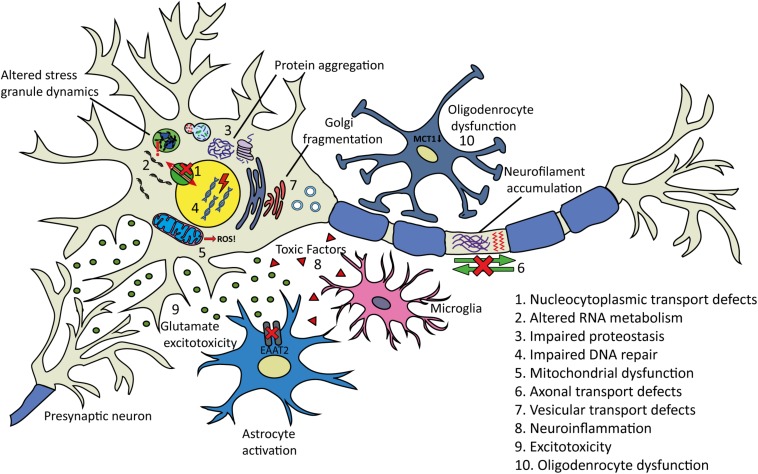
Proposed pathogenic mechanisms and pathology in ALS. (1) Nucleocytoplasmic transport defects including altered transport of RNA molecules and RNA-binding proteins. (2) Altered RNA metabolism. RNA-binding proteins including TDP-43 or FUS may become mislocalized in the cytoplasm leading to altered transcription and splicing. Stress granule dynamics are also affected. (3) Proteostasis is impaired with aggregating proteins including TDP-43 accumulating in the cytoplasm. There is evidence that the two main protein clearance pathways, autophagy and the UPS may be involved. (4) Impaired DNA repair: several ALS-linked genes including *FUS*, *TARDBP*, *TAF15*, *SETX*, and *EWSR1* are involved in DNA repair. (5) Mitochondrial dysfunction resulting in the increased formation of reactive oxygen species (ROS) has been proposed as an initiating factor in ALS. Several ALS-linked proteins including SOD1, TDP-43, and FUS interact with mitochondria. (6) Axonal transport defects have been implicated in ALS pathogenesis. Neuropathological evidence has shown evidence of this including neurofilament accumulation and cytoskeletal disorganization. (7) Several ALS-linked genes including *OPTN*, *VAPB*, *CHMP2B*, and *UNC13A* are involved in vesicular transport. Impaired vesicular trafficking can lead to protein accumulation and golgi fragmentation which has been observed in ALS patients. (8) Neuroinflammation: the secretion of inflammatory proteins by activated microglia leads to the potentially neurotoxic activation of astrocytes, which may contribute to the death of neurons and oligodendrocytes. (9) Excitotoxicity: glutamate receptor overstimulation has been proposed to occur via several mechanisms including increased synaptic glutamate release, alterations to AMPA receptors and reduced clearance of glutamate by astrocytes. (10) Oligodendrocyte dysfunction may lead to reduced support for neurons. Changes in lactate production and transport via MCT1 have been implicated.

Despite decades of research, causative pathogenic mechanisms in ALS still remain unclear, especially in sporadic cases. It is likely that multiple factors, rather than a single initiating event contribute to the development and progression of the disease. Furthermore, genetic and phenotypic variation between patients makes it difficult to uncover and draw conclusions regarding pathogenic mechanisms of ALS in general. The large number of genes and cellular processes implicated in ALS has led to the proposal of many disease mechanisms operating (Figure 4). These include disturbances in RNA metabolism, impaired protein homeostasis, nucleocytoplasmic transport defects, impaired DNA repair, excitotoxicity, mitochondrial dysfunction, oxidative stress, axonal transport disruption, neuroinflammation, oligodendrocyte dysfunction, and vesicular transport defects. Clarification is needed on the timing and extent to which each of these mechanisms contributes to disease pathogenesis.

### Altered RNA Metabolism

There was a shift in focus to RNA dysregulation as a key pathomechanism in ALS with the identification of disease-causing variations in RNA-binding protein genes, *TARDBP* and *FUS.* RNA-binding proteins are involved in several aspects of RNA metabolism, including splicing, transcription, transport, translation, and storage in stress granules. The number of RNA-binding proteins directly involved in neurodegeneration has grown to include *EWS* and *TAF15* (involved in FTD) (Neumann et al., 2011) *hnRNPA1* (Kim et al., 2013), and *MATR3* (Johnson et al., 2014b). A variety of RNA binding factors are found sequestered in association with the hexanucleotide repeat expansion in the *C9ORF72* gene transcript (Lee et al., 2013; Mori et al., 2013; Cooper-Knock et al., 2014). These discoveries have led to an increased interest in the role of RNA metabolism in neurodegenerative diseases.

Interestingly, many of the ALS-linked RNA-binding proteins contain prion-like domains that are involved in stress granule formation or dynamics, including TDP-43, FUS, TAF15, ESWR1, hnRNPA1, and hnRNPA2B1. Stress granules are transient RNA/protein complexes formed under cellular stress that are able to sequester specific mRNAs and prevent their translation. These granules facilitate cell survival by the translational arrest of non-essential transcripts and pro-apoptotic proteins when under stress (Protter and Parker, 2016). Prion-like domains are thought to be vital for the reversible assembly of stress granules due to their capacity for forming multiple transient weak interactions (Harrison and Shorter, 2017). Interest in the involvement of stress granules in ALS pathogenesis has recently increased.

### Nucleocytoplasmic Transport Defects

The mislocalization to the cytoplasm and nuclear depletion of RNA-binding proteins such as TDP-43 and FUS in ALS pathology suggest nuclear transport defects as a mechanism contributing to ALS pathogenesis. Recent studies into the consequences of the *C9ORF72* hexanucleotide repeat expansion have contributed to the growing evidence of nucleocytoplasmic transport defects in ALS. In a genetic screen in Drosophila to identify modifiers of C9ORF72 toxicity, 18 genetic modifiers involved in nucleocytoplasmic transport and RNA export were identified, highlighting this system as a primary target of hexanucleotide repeat expansion related toxicity (Freibaum et al., 2015). For a review of the role of nucleocytoplasmic transport in ALS see (Kim and Taylor, 2017).

### Impaired Proteostasis

The accumulation of damaged proteins contributes to several neurodegenerative diseases including Alzheimer’s, Huntington’s and Parkinson’s diseases, and has also emerged as a key characteristic in ALS. The most commonly mutated ALS genes (*SOD1*, *C9ORF72*, *TARDBP*, and *FUS*) all give rise to proteins that are found to aggregate in the neurons of ALS patients. Although protein aggregation is central to ALS pathology, questions remain about the formation, role and toxicity of these aggregates.

There is evidence that disruption of the two main protein clearance pathways, autophagy and the Ubiquitin-proteasome system (UPS) are involved in ALS pathogenesis. Autophagy is an intracellular pathway involved in the degradation and recycling of long-lived proteins and cytoplasmic organelles and is important in maintaining homeostasis in multicellular organisms (Kelekar, 2006). In the UPS pathway, proteins are marked for degradation by ubiquitination before being recognized and degraded by the proteasome (Finley, 2009). Several ALS associated genes encode proteins involved in autophagy or the UPS including *C9ORF72*, *OPTN*, *SQSTM1*, *VCP*, and *UBQLN2* (Majcher et al., 2015; Ying and Yue, 2016; Nassif et al., 2017). Given the role of ubiquitination in marking proteins for degradation, it is not surprising that pathological protein inclusions in ALS are ubiquitin-positive. Ubiquitination may be a critical factor in keeping TDP-43 and other protein levels in neurons within a healthy physiological range and the persistence of ubiquitin-positive aggregates is suggestive of impaired or overwhelmed protein clearance systems.

### Impaired DNA Repair

Impairment of DNA repair is another suggested mechanism that may contribute to ALS pathogenesis. Two of the best studied ALS linked proteins, TDP-43 and FUS, function in the prevention or repair of transcription-associated DNA damage (Hill et al., 2016). FUS in particular seems to play an important role in this regard and is involved in the repair of double-stranded DNA breaks via both homologous recombination and non-homologous end-joining repair mechanisms (Mastrocola et al., 2013; Wang W.-Y. et al., 2013). Variations in the genes of other ALS-linked RNA-binding proteins, including *TAF15*, *SETX*, and *EWSR1* have also been linked to impaired DNA damage repair, further implicating the breakdown of this process in ALS pathogenesis (Paronetto et al., 2011; Skourti-Stathaki et al., 2011; Izhar et al., 2015).

### Mitochondrial Dysfunction and Oxidative Stress

Oxidative stress has been suggested as a primary initiating factor in ALS pathogenesis. Oxidative stress occurs when a cell’s antioxidant capacity is superseded by its rate of production of reactive oxygen species. When antioxidants do not neutralize reactive oxygen species, they can seriously damage macromolecules such as DNA, proteins and phospholipids. There is morphological evidence of oxidative damage, including lipid peroxidation and protein glycoxidation in the spinal cords of sALS patients (Shibata et al., 2001).

Mitochondria produce the majority of reactive oxygen species with large amounts of superoxide radicals generated as a by-product of cellular respiration, leading researchers to investigate potential mitochondria-related pathogenic mechanisms in ALS. These have included the disruption of mitochondrial function by aggregated products of ALS related genes, impaired clearance of damaged mitochondria by autophagy and dysfunction and mitochondrial damage due to aberrant RNA processing (Carrì et al., 2017). The role of SOD1 as a cytosolic anti-oxidative enzyme was also suggestive of a role for oxidative stress in ALS pathogenesis. Interestingly, mutant SOD1 is not generally thought to cause disease through loss of its antioxidative function, but rather through interactions that upregulate reactive oxygen species as well as through altered interactions with mitochondria and other proteins (Cozzolino et al., 2009).

Both oxidative stress and RNA misregulation occur in ALS pathogenesis and there are dual views regarding mechanism and causation. One hypothesis is that oxidative stress may cause RNA dysregulation. There is some evidence for this with oxidative stress shown to cause mislocalization and increased aggregation tendency of regulatory RNA proteins TDP-43 and FUS (Vance et al., 2013; Cohen et al., 2015). Others have suggested that RNA dysregulation may be causative of oxidative stress and mitochondrial damage that could occur via several mechanisms. Damage may be induced through direct interaction of ALS associated proteins with mitochondria, leading to increased formation of reactive oxygen species (Pickles et al., 2016). Altered interactions of RNA-binding proteins such as TDP-43 and FUS with non-mitochondrial proteins may also contribute to mitochondrial dysfunction. FUS, for example, is thought to localize in the mitochondria through interactions with the mitochondrial chaperone protein HSP60 (Deng et al., 2015). Yet another possibility is that pathogenic RNA binding proteins in ALS, such as TDP-43 may directly affect the regulation of mRNA coding for proteins involved in mitochondrial physiology. There is evidence for this hypothesis with overexpression, underexpression or mutations in TDP-43 shown to influence mitochondrial dynamics, causing dysfunction (Wang W. et al., 2013).

### Axonal Transport Defects

Axonal transport involves the movement and spatiotemporal distribution of intracellular cargo such as lipids, proteins, mRNA, membrane-bound vesicles, and organelles along the axon. It is important in maintaining the structure and function of the cell and in the long-distance communication between the cell body and synaptic terminals. Axonal transport defects are commonly seen in neurodegenerative diseases (Millecamps and Julien, 2013). Early neuropathological evidence consistent with axonal transport defects in ALS came from post-mortem studies. These revealed abnormal accumulations of neurofilaments, mitochondria and lysosomes as well as spheroids containing vesicles, lysosomes, mitochondria, neurofilaments, and microtubules (Hirano et al., 1984a, b; Corbo and Hays, 1992; Rouleau et al., 1996; Sasaki and Iwata, 1996). Axonal transport was later found to be impaired in several *SOD1* mouse models (Zhang et al., 1997; Williamson and Cleveland, 1999; Ligon et al., 2005; Bilsland et al., 2010). Pathogenic variants in axonal transport machinery and cytoskeletal genes such as those in *TUBA4A* have since been indicated as a primary cause of ALS in rare cases (Smith et al., 2014).

There is evidence to suggest that TDP-43 may be involved in axonal transport defects seen in ALS. TDP-43 facilitates the delivery of mRNA via active axonal transport (Akira et al., 2016), and pathogenic variants in TDP-43 impair this movement in Drosophila, mouse cortical neurons and in ALS patient stem cell-derived neurons (Alami et al., 2014). In mouse models, axonal transport defects were observed early in disease progression, before symptom onset, suggesting a role in pathogenesis (Williamson and Cleveland, 1999).

### Vesicular Transport Defects

Several proteins involved in vesicular transport have been linked to ALS and FTD, implicating defective vesicular transport in ALS pathogenesis. These include *OPTN*, *VAPB*, *CHMP2B*, and *UNC13A* (Cox et al., 2010; Urwin et al., 2010; Vidal-Taboada et al., 2015; Chadi et al., 2017; Sundaramoorthy et al., 2017). Prolonged inhibition of vesicular trafficking from the golgi to the plasma membrane can lead to protein accumulation and golgi fragmentation (Zolov and Lupashin, 2005; Zhou Z. et al., 2013; Sundaramoorthy et al., 2015), the latter being a prominent histopathological feature seen in ALS patients (Gonatas et al., 1992; Mourelatos et al., 1994; Fujita and Okamoto, 2005; Fujita et al., 2007). Evidence indicates that fragmentation of the golgi apparatus occurs early in the pathological cascade of disease, suggesting that it may be a trigger of neurodegeneration, rather than a consequence (Gonatas et al., 2006; Dis et al., 2014).

### Neuroinflammation

There is increasing evidence that neuroinflammation plays a role in ALS pathophysiology. Neuroinflammation associated with neuronal loss is characterized by microglia and astrocyte activation, overproduction of inflammatory cytokines and infiltration of T lymphocytes (Komine and Yamanaka, 2015). There is strong evidence for the activation of microglia in ALS (Turner et al., 2004; Corcia et al., 2012). Microglia are able to detect insults and injuries that disrupt brain homeostasis, responding with a change in morphology and the release of cytokines and chemokines to clear pathogens or debris. The secretion of inflammatory proteins by activated microglia leads to the potentially neurotoxic activation of astrocytes that may contribute to the death of neurons and oligodendrocytes (Liddelow et al., 2017). Astrocytes provide trophic support for neurons, prune synapses and provide other homeostatic functions in the CNS (Clarke and Barres, 2013). The potential of microglia to play a direct role in ALS pathogenesis was highlighted by the identification of several ALS linked genes that influence the function of, and are highly expressed in microglia, including *C9ORF72*, *TBK1*, and *PGRN* (Irwin et al., 2008; Freischmidt et al., 2015; Lall and Baloh, 2017). Immune cells have been implicated both in protective effects on neuronal survival and in exerting deleterious effects, depending on the stage of disease progression (Liu and Wang, 2017).

### Excitotoxicity

Excitotoxicity is a pathological process of glutamate receptor overstimulation resulting in neuronal damage or degeneration (Dong et al., 2017). Glutamate acts as a neurotransmitter and is released from presynaptic terminals to act on post- and pre-synaptic glutamate receptors. In the CNS, extracellular glutamate levels are kept low with intracellular levels being much higher. When the extracellular glutamate level is elevated, neurons are damaged through excessive stimulation of glutamate receptors. Excitotoxicity has long been suspected as a mediator of disease in ALS. Oral intake of excitotoxins is responsible for particular forms of motor neuron disease, suggesting motor neurons may be particularly vulnerable to excitotoxicity (Spencer et al., 1986). Current evidence for altered glutamate levels in ALS patients is contradictory with results varying across studies (Van Cutsem et al., 2006). The evidence for altered glutamatergic signaling in ALS comes primarily from electrophysiological studies (de Carvalho et al., 2017).

There are several mechanisms by which the dysregulation of glutamatergic transmission has been proposed to occur. Direct mechanisms include the increased synaptic release of glutamate or insufficient re-uptake from the synaptic cleft. Alterations in glutamate receptor expression or function including alterations to AMPA receptors leading to increased influx of Ca^2+^ has also been proposed (Kwak et al., 2010). Reduced expression and activity of glutamate receptor EAAT2 on glial astrocytes may also contribute to excitotoxicity via reduced glutamate clearance (Boston-Howes et al., 2006). Other hypotheses of excitotoxic causality include altered regulation by interneuron populations, intrinsic excitability of motor neurons or the release of intracellular glutamate from lethally damaged neurons, astrocytes or microglia. Much research has gone into the study of these mechanisms in ALS with mixed conclusions, as reviewed by King et al. (2016). The question remains as to whether altered glutamatergic signaling in ALS patients is directly pathogenic or whether it is driven by other factors. While the use of Riluzole has shown modest benefit to some ALS patients by addressing excitotoxicity, numerous other drugs targeting glutamatergic transmission have been unsuccessful in clinical trials.

### Oligodendrocyte Dysfunction

There is growing evidence supporting the contribution of non-neuronal cells, such as oligodendrocytes, to ALS pathogenesis. Oligodendrocytes are the myelinating cells in the CNS responsible for producing the myelin sheath that insulates the axons of nerves and neurons (Bradl and Lassmann, 2010). Gray matter oligodendrocytes are not involved in myelin sheath formation and are thought to play a role in providing metabolic support to neurons (Nave, 2010; Philips and Rothstein, 2017).

Oligodendrocytes are proposed to contribute to axonal degeneration through changes in the production of the metabolite lactate. There is some evidence for this with the dominant lactate transporter in the brain, MCT1, that is highly enriched within oligodendroglia, reduced in the motor cortex in mouse models of ALS and in ALS patients (Lee et al., 2012). SOD1^G93A^ transgenic mice also have impaired function and extensive degeneration of gray matter oligodendrocytes in the spinal cord, with oligodendrocyte precursors failing to fully differentiate (Kang et al., 2013). It remains unclear whether these changes precede neuronal loss or are a consequence of it. Alterations in myelin structure in the spinal cords of mice occur presymptomatically and early in disease progression indicating possible contributions to causality (Niebroj-Dobosz et al., 2007; Kang et al., 2013).

## Approaches to Treatment

### History

The current standard of care for ALS involves multidisciplinary symptom management including nutritional and respiratory support. The most widely used treatment for ALS is Riluzole. This drug was first approved in 1995 and is only mildly effective, prolonging survival for up to 2–3 months in some patients and exerting a beneficial effect only in the first 6 months of therapy (Bensimon et al., 1994; Cetin et al., 2015). When launched, Riluzole was hypothesized to act via the modulation of glutamatergic transmission (Bensimon et al., 1994). Research into the mechanism of action of Riluzole has consistently shown that its effects on glutamate receptors are limited, and that the mechanisms of action are likely more varied and complex (Bellingham, 2011). This may help to explain why other anti-glutamatergic compounds such as Ceftriaxone, Memantine, and Talampanel have failed to show efficacy in ALS clinical trials (de Carvalho et al., 2010; Pascuzzi et al., 2010; Cudkowicz et al., 2014).

During the years since Riluzole was launched, over 60 other molecules have been investigated as potential treatments for ALS (Petrov et al., 2017). Treatments reaching clinical trials have overwhelmingly been anti-inflammatory, anti-oxidative, anti-glutamatergic, neuroprotective, and neurotrophic compounds. The most commonly used primary outcome measure to evaluate the efficacy of treatments in ALS is the ALSFRS-R scale. Scores on the scale are arrived at through a 12-question form that evaluates the gross motor, fine motor, bulbar, and respiratory function of patients (Cedarbaum et al., 1999). The vast majority of compounds tested have failed to demonstrate efficacy in human clinical trials, most of which measured differences in ALSFRS-R scores or survival time.

In 2017, the first new treatment for ALS in over two decades was approved for use in Japan, South Korea and the United States. Radicava (Edaravone) is an antioxidative compound proposed to function by reducing oxidative stress, although its exact mechanism is unknown. Edaravone was initially approved for use in Japan for the treatment of cerebral embolism. Efficacy of Edaravone for the treatment of ALS was tested in two phase III clinical trials and was determined by the change in ALSFRS-R scores compared to baseline. The first double-blind placebo-controlled trial (MCI-186-16) reported no significant differences between treatment and placebo groups (Abe et al., 2014). After a *post hoc* analysis of the results was carried out, a follow-up study (MCI-186-19) in a more narrowly defined patient population was undertaken. This trial reported a modest but statistically significant difference in ALSFRS-R scores, with Edaravone treated patients showing reduced functional loss when compared to patients receiving a placebo after 6 months of treatment (Sawada, 2017). Concomitant use of Riluzole was allowed during this study. The majority of treatments investigated for ALS thus far have been small molecules. Although success has been limited, there is hope that alternative approaches, including RNA based therapeutics may be more fruitful.

### Molecular Approaches to Treat ALS

RNA targeted therapeutics have entered a new phase of growth with the two main strategies investigated being short interfering RNA (siRNA) and antisense oligonucleotides (AOs). siRNA are double-stranded RNA molecules that can be used to downregulate the expression of target genes to which they are complementary through interactions with the RNA-induced silencing complex. Although there have been several preclinical investigations on the use of siRNA to target ALS genes, none have yet reached clinical trials (Ding et al., 2003; Nishimura et al., 2014). This review focuses on potential AO therapeutics for ALS. AOs are short, single-stranded nucleic acids that can bind to RNA through Watson-Crick base pairing and can alter gene expression through several different mechanisms. They can be used to restore or reduce protein expression or to modify protein isoform production through splice switching strategies.

Therapeutic use of many RNA analog drugs has been slowed by inefficient and poorly targeted delivery. Unmodified single-stranded RNA is unable to cross the cell membrane efficiently unaided, due to its size and negative charge and is susceptible to rapid degradation by nucleases. A range of chemical modifications has helped to address some of these issues, as reviewed by Khvorova and Watts (2017). Synthetic RNA-like drugs are commonly delivered to target cells using a nanoparticle delivery platform (usually a cationic polymer or lipid) or through conjugation to a bioactive ligand or cell penetrating peptide (Kaczmarek et al., 2017). Achieving effective concentrations of AOs in the organ or tissue of interest can be challenging, although treatment of neurodegenerative diseases with AOs may be advantageous in this regard as the AOs can be administered directly to the CNS via intrathecal administration. Once in the nervous system, AOs are readily taken up by neurons and glia (Smith et al., 2006). Furthermore, the blood-brain barrier prevents the dispersion into peripheral tissues and subsequent clearance by the kidney and liver, allowing clinically effective concentrations to be more easily reached. This means smaller doses can be used, minimizing any potential toxicity (Evers et al., 2015) or systemic off-target effects. Several AO drugs have received FDA approval in recent years to treat a variety of conditions, including Eteplirsen (Exondys 51^®^) for Duchenne muscular dystrophy (Stein, 2016) Nusinersen (Spinraza^®^) for spinal muscular atrophy (Singh et al., 2017) and Inotersen (Tegsedi^®^) for hereditary transthyretin-mediated amyloidosis (hATTR) (Keam, 2018). Exploration of AO therapeutics has also begun for the treatment of ALS.

The first AO drug in clinical development for the treatment of ALS was developed by ISIS Pharmaceuticals (now Ionis) and targets the *SOD1* transcript (Miller et al., 2013). This AO exploits RNase H mediated degradation to target both mutant and wild-type *SOD1* mRNA. As the toxicity of SOD1 is proposed to be primarily due to a gain of function rather than a loss of function, knockdown of the protein is not expected to be harmful (Miller et al., 2013). Phase I studies to evaluate the safety, tolerability, and pharmacokinetics are ongoing (NCT02623699). AOs targeting the *SOD1* mRNA that utilize different chemistries and with alternative mechanisms of action are also in preclinical development by several groups.

Pre-clinical studies of AOs targeted to *C9ORF72* transcripts have also begun. In the case of C9ORF72, researchers have been able to exploit the location of the pathogenic hexanucleotide expansion that occurs in an intron between two alternatively used first exons. AOs utilizing RNase H mediated degradation that targets only HRE containing transcripts or all C9ORF72 transcripts have been developed with both methods reported to reduce RNA foci in patient-derived fibroblasts and iPSCs (Donnelly et al., 2013; Lagier-Tourenne et al., 2013; Sareen et al., 2013). HRE targeting AOs have also been able to reduce pathological *C9ORF72* RNA in transgenic mice (Jiang et al., 2016).

There are currently no AOs in clinical development that directly target the *TARDBP* or *FUS* transcripts. AOs that target *ATXN2* transcripts have been tested in TDP-43 transgenic mouse models (Becker et al., 2017). Ataxin-2 is thought to promote the maturation of stress granules (Kaehler et al., 2012), and reduced levels were hypothesized to decrease the recruitment of TDP-43 into these granules, potentially reducing the propensity of TDP-43 to form pathologic inclusions. The AOs reduced TDP-43 aggregation, extended lifespan and reduced pathology in the *TDP-43^Tg/Tg^* mouse model.

With the scope of RNA therapeutics rapidly expanding and the genetic basis of ALS continuing to be uncovered, AOs may be a promising area for future therapeutic developments for subsets of ALS patients.

## Models for Als

Determining the mechanisms involved in ALS and other brain disorders has been challenging due to the difficulty in obtaining living tissue or cells from the CNS of patients. A wide variety of model systems have been utilized by researchers to investigate the complex processes occurring in this disease. These models vary widely and include *in vitro* biochemical systems, cell lines and primary cell cultures, various small animal and rodent models. More recently, patient-derived cellular models are being developed and evaluated.

### Rodent Models

Nearly all human genes have a homolog in the mouse and rat. Evaluation of drugs in these animals therefore increases the chances of successful translation of results to humans over lower animal models. Rodent models have been used extensively in ALS research since the first *SOD1*^(G93A)^ and *SOD1*^(A4V)^ ALS mouse models were produced in 1994 (Gurney et al., 1994). Yet for therapeutic applications, translation from animal models to successful outcomes in human clinical trials has remained dismal. Several factors have contributed to this outcome; overall, rodents generally display milder ALS phenotypes than humans, with animals in some models showing no signs of neurodegeneration. The shorter life-span of rodents means that pathological changes have a shorter time to develop when compared to humans and confounding effects may also be conferred by the genetic background of the rodent. These factors may contribute to the differences in pathology and milder symptoms, potentially limiting the usefulness of rodent models in studying the late stages of neurodegenerative disease. There are also significant differences in RNA splicing and metabolism between humans and rodents. Although there is a high rate of conservation for constitutive exons between humans and rodents, for minor forms and tissue specific transcripts, only about a quarter of alternatively spliced exons are conserved between human and rodents (Modrek and Lee, 2003). The significance of this difference in regard to ALS pathogenesis remains unexplored. Physiological differences between humans and rodents also exist including differences in cellular and molecular functionality, as well as gross neuroanatomy and circuitry (Bodenreider et al., 2005). These factors have likely contributed to the overwhelming failure of translation from pre-clinical animal studies to effective clinical therapies for ALS. Comparisons of pathology between human ALS and some ALS rodent models are reviewed below.

Several transgenic *SOD1* rodent models have been developed in the past 25 years, most of which overexpress missense, mutant or truncated human *SOD1*. The *SOD1*^(G93A)^ model however remains the most widely utilized. *SOD1* rodent lines generally develop adult-onset motor neuron disease, reminiscent of the human disease with hallmarks of human disease such as SOD1 aggregation, excitotoxic cell death of neurons, neuroinflammatory reactions and altered oligodendrocyte biology replicated in *SOD1* mouse models (Philips and Rothstein, 2015). These models have been used to characterize ALS biology and in preclinical evaluation for potential therapeutics. Although several potential therapeutics have been able to affect disease and show benefit in *SOD1* mouse models, translation to benefit in human clinical trials has been poor. Minocycline, for example, was able to slow disease in *SOD1*^(G37R)^ mice (Kriz et al., 2002) but was shown to accelerate disease in human clinical trials involving a diverse ALS patient group (Gordon et al., 2007).

TDP-43 rodent models, in contrast, often show significant differences in disease phenotype from that in humans. Most TDP-43 mouse models do not appear to develop ALS symptoms similar in severity to humans (Philips and Rothstein, 2015) and in low-level overexpression models, loss of cortical or spinal motor neurons is modest compared to that in human ALS patients (Philips and Rothstein, 2015). Another notable lack or correlation between rodent models and humans is the presence of TDP-43 positive cytoplasmic inclusions that, while widely reported in human ALS, are not regularly seen in rodent models of disease (Arnold et al., 2013).

FUS rodent models have been developed that include knockout, knockdown and overexpression models of both wild-type and mutated FUS proteins. While providing clues as to the pathomechanism of FUS-ALS, no models to date have been able to reproduce the distinct neuropathological features seen in humans (Nolan et al., 2016). Furthermore, phenotypic discrepancies between models are common; several transgenic models using the same R521C mutation, for example, have been produced with a range of phenotypes reported (Huang et al., 2011; Verbeeck et al., 2012; Qiu et al., 2014; Sharma et al., 2016).

Rodents modeling the *C9ORF72* hexanucleotide repeat expansion have recently been developed. A C9ORF72 mouse model that expresses 66 repeats in the CNS, mediated by adeno-associated virus was established, with mice developing histopathological and clinical symptoms of ALS, including motor deficits (Chew et al., 2015). In contrast, two independent mouse models that express the human hexanucleotide repeat expansion from a bacterial artificial chromosome were generated (O’Rourke et al., 2015; Peters et al., 2015); in both cases, mice developed the distinctive histopathological features seen in human ALS, including widespread RNA foci and dipeptide repeat proteins, however, no behavioral changes or neurodegeneration were observed. This supports the hypotheses that RNA foci and dipeptides are not sufficient to drive degeneration, although it is possible that these results are specific to these models and are not representative of human disease.

Another mouse model widely used in ALS research is the “wobbler” mouse. The wobbler mouse arose spontaneously in a C57BL/Fa strain and was first described by Falconer in 1956 (Falconer, 1956). These mice display phenotypic features similar to human ALS including muscle weakness, motor defects and a marked loss of motor neurons. Wobbler mouse motor neurons share many of the characteristics described in human ALS including vesicle transport defects, mitochondrial dysfunction, enlarged endosomes, vacuolization, impaired axonal transport, cortical excitotoxicity, ubiquitin-positive protein aggregates and neurofilament aggregation (Moser et al., 2013). TDP-43 pathology in the wobbler mouse resembles that seen in sporadic human ALS, including elevated TDP-43 levels, abnormal distribution to the cytoplasm, the presence of carboxy-terminal fragments and co-localization with ubiquitin-positive inclusions (Dennis and Citron, 2009). The variant responsible for this phenotype was uncovered in 2005 as a point mutation in the *VPS54* gene (Drepper et al., 2005). The VPS54 protein is part of the Golgi-associated retrograde protein complex with variations in this gene resulting in impairment of retrograde vesicle transport (Pérez-Victoria et al., 2010). Although no ALS-linked variants in the homologous human gene have yet been described, this model has been used to test a variety of potential ALS therapeutics over the years, including trophic neuroprotective factors, anti-inflammatory agents, anti-glutamatergic agents, mitochondrial support agents, antioxidants, steroids and stem cell therapies as reviewed by Moser et al. (2013).

### Higher Mammal Models

Although not widely used, alternative animal models for ALS, including pigs and primates, are being explored. The similarities between pigs and humans make pigs a valuable model in disease characterization and therapy development and they have, therefore, been used to model TDP-43 and SOD1-ALS. Transgenic pigs expressing the TDP-43 M337V variant show a severe ALS like phenotype and the cytoplasmic mislocalization of TDP-43, but not the formation of aggregates (Wang et al., 2015). *SOD1*^(G93A)^ pigs showed nuclear accumulation of SOD1 (Yang et al., 2014) with both TDP-43 and SOD1 models uncovering protein interactions not seen in rodent models of the disease. Primate models are also shedding light on disease pathomechanisms. In a TDP-43 overexpression model that closely recapitulates ALS phenotypes seen in humans, TDP-43 was found to be mislocalized early in disease progression. In this model, TDP-43 phosphorylation was only detected in the later stage of disease and the signature 25-kDa C-terminal fragment was not detected, indicating these may not be necessary to initiate TDP-43 induced neuronal dysfunction in monkeys (Uchida et al., 2012). In a recent study that compared transgenic monkeys and mice expressing the TDP-43 M337V variant, species dependent localization and cleavage of TDP-43 was apparent. Interestingly, the C-terminal fragments were abundant in the monkeys’ brains in this model, in contrast to the TDP-43 overexpression monkey model (Yin et al., 2019).

There is no doubt that animal models have been useful in helping uncover mechanisms of pathogenesis in ALS, but it is important to also recognize their limitations. No animal model is able to fully replicate the entire spectrum of phenotypes seen in human ALS. The heterogeneity of ALS may lead to varied responses to similar treatments in different patients. This makes it difficult to assess how a therapeutic effect may translate from animal models to efficacy in humans. Animal models clearly need to be supplemented with pre-clinical models that can capture the heterogeneity of disease seen in humans.

### iPSCs

Recently, the use of patient-derived stem cell models has emerged in ALS research; many of the first investigations have used iPSC derived motor neurons. iPSCs are reprogrammed from somatic cells by the introduction of key pluripotency factors. This was first achieved in mouse fibroblasts in 2006 (Takahashi and Yamanaka, 2006) and in human cells the following year (Takahashi et al., 2007). The pluripotent cells can then be guided to differentiate into the desired cell lineage including neurons, motor neurons or glial cells. This approach provides several advantages over other cellular models for the study of human genetic diseases. Firstly, the need to overexpress transgenes containing pathogenic gene variants is eliminated. Additionally, these cells carry endogenous gene variants within the context of an individual’s genetic background making them particularly valuable for the study of sporadic disease in which the causative genetic factors are unknown. iPSC-derived neurons have proved to be a valuable model for ALS, with several aspects of disease neuropathology recapitulated. Motor neurons derived from iPSCs have been utilized to study variants in *TARDBP*, *C9ORF72*, *SOD1*, *FUS* and in models of sporadic disease.

Various ALS-associated *SOD1* variants have been modeled using iPSCs. These models have the capacity to reflect pathology seen in patients, such as SOD1 aggregation and have been used to explore disease mechanisms (Bhinge et al., 2016; Seminary et al., 2018). Whilst iPSC derived motor neurons from patients with pathogenic *TARDBP* variants were reported to show characteristics of TDP-43 proteinopathy, results have not always been consistent. Increased levels of soluble and detergent resistant TDP-43 and decreased cell survival were reported in a *TARDBP* M337V iPSC model for example (Bilican et al., 2012; Egawa et al., 2012), whilst others reported no difference in cytoplasmic TDP-43 aggregation or cell survival between TARDBP mutant iPSC motor neurons and controls, despite the same variant (M337V) (Seminary et al., 2018). iPSC-derived motor neurons from patients with pathogenic *FUS* variants develop typical FUS pathologies, including the cytoplasmic mislocalization of FUS and recruitment into stress granules. These models have also been useful in demonstrating the relationship between specific point mutations and cytosolic FUS mislocalization (Japtok et al., 2015; Lenzi et al., 2015; Ichiyanagi et al., 2016; Guo et al., 2017). iPSC derived motor neurons from *C9ORF72* ALS patients harbor disease-associated characteristics, including aggregation of mRNA containing the hexanucleotide repeat expansion and the formation of RAN translated dipeptides (Almeida et al., 2013). *C9ORF72* iPSC derived motor neurons have revealed other disease characteristics associated with the *C9ORF72* hexanucleotide repeat expansions such as alterations in gene expression, nucleocytoplasmic transport defects and susceptibility to excitotoxicity (Donnelly et al., 2013; Sareen et al., 2013; Freibaum et al., 2015). Although iPSC derived motor neurons have proved valuable in ALS research, there is also valid concern over genetic and epigenetic variations in iPSCs that may compromise their utility (Liang and Zhang, 2013). As aging is the leading risk factor in the development of ALS and other neurodegenerative disorders, it is important to consider the age-related characteristics of the cells being studied. iPSC derived neurons do not maintain the aging and epigenetic signatures of the donor (Hewitt and Garlick, 2013).

### Directly Reprogrammed Motor Neurons

Although not yet in widespread use in ALS research, an alternative approach to generating patient derived motor neurons that more acutely model the age-related phenotype is gaining momentum. Fully differentiated somatic cells are now able to be directly reprogramed into functional neurons that may be guided to subtype-specific neurons, based on the addition of specific transcription factors or microRNAs (Pang et al., 2011; Yoo et al., 2011). This method has been used to successfully convert fibroblasts from ALS patients with both pathogenic *FUS* variants and *C9ORF72* expansions to motor neurons that displayed disease specific degeneration (Su et al., 2014; Lim et al., 2016; Liu et al., 2016). It has been shown that directly reprogrammed motor neurons are able to maintain the aging hallmarks of old donors, including heterochromatin loss, DNA damage and nuclear organization (Tang et al., 2017). Although only beginning to be explored in ALS research, directly reprogrammed motor neurons may be more suitable than iPSC derived motor neurons to model late-onset neurodegeneration.

### Olfactory Stem Cell Cultures

Another patient-derived stem cell model that has recently emerged in the study of brain diseases uses stem cells derived from the olfactory mucosa of human patients. The olfactory mucosa is a neural tissue that is easily accessible in a clinical setting (Féron et al., 1998) and contains several cell types including a large population of multipotent neural stem cells. These stem cells can be purified from surrounding cells and expanded as neurospheres (clusters of neural progenitor cells) that may then be propagated in neurospheres as neural progenitor cells or dissociated and propagated as olfactory neurosphere-derived stem cells (ONS cells) (Féron et al., 2013). ONS cells may also be differentiated to form neurons or glia for further study.

Recent advances have made less invasive approaches to tissue biopsy available, with olfactory stem cells now readily obtainable from living human subjects (Benítez-King et al., 2011). Improvements in the enrichment of neural cells from biopsied tissue have also occurred (Tajinda et al., 2010), with several advantages in using olfactory mucosa-derived stem cells over iPSCs. Firstly, the reprogramming step from somatic cell to stem cell is bypassed, reducing confounding events such as epigenetic changes that can interfere with accurate assessment of differential gene expression. In addition, elimination of the re-programming step makes the use of olfactory derived cells less time consuming and less costly, making this cell type more suitable for high throughput studies. Furthermore, ONS cells can be easily maintained in culture and are suitable for use in multiwell assays, making them useful for many applications, including understanding disease etiology, diagnostics, and drug screening.

Patient-derived ONS cells have shown disease-specific alterations in gene expression and cell function in several complex neurological disorders including schizophrenia, Parkinson’s disease, and dysautonomia (Boone et al., 2010; Cook et al., 2011; Fan et al., 2012; Mackay-Sim, 2012). ONS cells have also demonstrated utility in modeling hereditary spastic paraplegia (Abrahamsen et al., 2013), an adult-onset disease involving axonal degeneration in corticospinal motor neurons and in ataxia-telangiectasia (Stewart et al., 2013), a rare disorder with varying symptoms including neuropathological features. ONS cells are a promising platform for gene expression studies, drug discovery and diagnostics in neurological disease and may be of utility in ALS research. ONS cells and ONS derived neurons may be particularly useful in modeling early disease processes, before disease onset and in modeling sporadic disease.

Although the use of patient derived motor neurons has been validated for use in exploring the molecular basis of ALS and developing new drug screening platforms, several challenges remain. In order to enable comparison of results across laboratories, it is necessary to develop and define standard criteria for the maturation, molecular characterization and electrical and transcriptional functioning of patient derived motor neurons (Sances et al., 2016). Most studies to date have represented only a few patients; increasing sample sizes in these studies will strengthen the observed ALS related phenotypes.

## Challenges and Future Directions

Many challenges remain for researchers in the pursuit of understanding and ultimately providing effective treatments for people living with ALS. Questions regarding unidentified heredity remain unanswered. Single nucleotide polymorphisms account for only a fraction of ALS cases. A shift in focus to genetic disease modifiers, such as copy number variations and structural variants as well as further interrogation of intronic DNA regions during DNA diagnosis may help to fill in the gaps.

It remains difficult to unravel the temporal etiology of ALS with downstream effects and potential causes feeding back into each other. The contribution that each of the proposed disease mechanisms plays in ALS pathogenesis and which of these are initiating factors remains unclear, with conflicting reports from researchers depending on the protocols and model systems used. Models that reflect early and even pre-symptomatic disease pathogenesis may help to discriminate initiating factors causative of disease from follow-on consequences. It is clear that TDP-43 is involved in ALS pathogenesis in most ALS cases. At what stage of disease progression it becomes involved still needs to be determined, as does the role of the various post-translational modifications to TDP-43 seen in ALS. The degree to which aggregation of ALS proteins mediates toxicity also requires further investigation.

Given the large number of genetic variants associated with ALS and the number of cellular pathways affected, there is likely a large amount of heterogeneity between patients. No animal model is able to fully replicate the spectrum of phenotypes seen in human disease making it very difficult to unravel the causes and determine effective treatments. Translation from benefit in animal models to benefit in humans remains poor; this is likely to have multiple causes, however, it is probable that pathophysiologic heterogeneity between patients is a major contributing factor. Utilizing patient-derived cellular models from large numbers of individuals with ALS may help in more accurately determining treatments that may be of broad benefit to patients.

Personalized medicine is a medical scheme that incorporates genetic, clinical diagnostics and environmental information to individualize patient care, and is gradually becoming more commonplace especially in the treatment of cancers. Personalized medicine is an important advancement as subtypes of patients may respond differently to potential disease-modifying therapies. The further identification of ALS patient subtypes according to genetic and non-genetic information and the identification of clinical biomarkers would be most beneficial. Taking complete genetic information into account when assessing clinical trial outcomes may help in determining treatments that are most suitable for particular subgroups of patients. An example demonstrating the benefit of this approach comes from a genetic *post hoc* meta-analysis on the data from three recent trials into the efficacy of lithium carbonate to treat ALS. Although no improvement in 12-month survival was reported in any of these studies, investigators found that those homozygous for a common A > C single nucleotide variant in the *UNC13A* gene had a significant increase in survival probability after 12 months; increasing from 40.1 to 67.7% after treatment (van Eijk et al., 2017).

Gene expression analysis has shown molecular heterogeneity exists in sALS patients with different pathways and genes dysregulated (Aronica et al., 2014). Ultimately, a comprehensive systems biology approach that integrates genomics and other cellular information with bioinformatic analyses could greatly improve our understanding of this complex and multifactorial disease and help in developing a more accurate biomarker-assisted diagnosis, and hopefully more personalized and effective treatment strategies.

## Author Contributions

RM: conception and initial draft writing. IP, LF, PA, SF and SW: critical editing of the manuscript.

## Conflict of Interest

The authors declare that the research was conducted in the absence of any commercial or financial relationships that could be construed as a potential conflict of interest.
